# Assessment of the Genetic Relationship and Population Structure in Oil-Tea Camellia Species Using Simple Sequence Repeat (SSR) Markers

**DOI:** 10.3390/genes13112162

**Published:** 2022-11-19

**Authors:** Heqin Yan, Huasha Qi, Yang Li, Yougen Wu, Yong Wang, Jianmiao Chen, Jing Yu

**Affiliations:** 1 Sanya Nanfan Research Institute of Hainan University, Hainan Yazhou Bay Seed Laboratory, Sanya 572025, China; 2 Key Laboratory for Quality Regulation of Tropical Horticultural Plants of Hainan Province, College of Horticulture, Hainan University, Haikou 570228, China; 3 Institute of Tropical Horticulture Research, Hainan Academy of Agricultural Sciences, Haikou 571100, China; 4 Engineering Research Center for the Selection and Breeding of New Tropical Crop Varieties of Ministry of Education, College of Tropical Crops, Hainan University, Haikou 570228, China; 5 Ministry of Education Key Laboratory for Ecology of Tropical Islands, College of Life Sciences, Hainan Normal University, Haikou 571158, China

**Keywords:** oil-tea camellia, SSR, genetic relationship, genetic diversity, population structure

## Abstract

Oil-tea camellia trees, the collective term for a class of economically valuable woody oil crops in China, have attracted extensive attention because of their rich nutritional and pharmaceutical value. This study aimed to analyze the genetic relationship and genetic diversity of oil-tea camellia species using polymorphic SSR markers. One-hundred and forty samples of five species were tested for genetic diversity using twenty-four SSR markers. In this study, a total of 385 alleles were identified using 24 SSR markers, and the average number of alleles per locus was 16.0417. The average Shannon’s information index (I) was 0.1890, and the percentages of polymorphic loci (P) of oil-tea camellia trees were 7.79−79.48%, indicating that oil-tea camellia trees have low diversity. Analysis of molecular variance (AMOVA) showed that the majority of genetic variation (77%) was within populations, and a small fraction (23%) occurred among populations. Principal coordinate analysis (PCoA) results indicated that the first two principal axes explained 7.30% (PC1) and 6.68% (PC2) of the total variance, respectively. Both UPGMA and PCoA divided the 140 accessions into three groups. *Camellia oleifera* clustered into one class, *Camellia vietnamensis* and *Camellia gauchowensis* clustered into one class, and *Camellia crapnelliana* and *Camellia chekiangoleosa* clustered into another class. It could be speculated that the genetic relationship of *C. vietnamensis* and *C. gauchowensis* is quite close. SSR markers could reflect the genetic relationship among oil-tea camellia germplasm resources, and the results of this study could provide comprehensive information on the conservation, collection, and breeding of oil-tea camellia germplasms.

## 1. Introduction

Oil-tea camellia trees is the collective term for a group of plants of high economic value. There are approximately 50 species of these trees, and they belong to the family Theaceae [1]. Oil-tea camellia trees have high value and a wide range of uses. They can be used as chemical bioenergetics, chemical feedstock, and a nutrient source [2,3]. Oil-tea camellia trees have a long history of cultivation in China and are mainly distributed in areas south of the Yangtze River Valley [4,5]. The main cultivated species are *Camellia chekiangoleosa*, *Camellia oleifera*, *Camellia crapnelliana*, *Camellia vietnamensis* Huang, etc. [5,6]. Nevertheless, the quality and oil yield of oil-tea camellia trees may vary depending on the species [3]. Thus, it is essential to form molecular markers for identification of populations or species to support breeding improvement and promote the development of genetic resources for oil-tea camellia trees.

Due to its extensive planting under different ambient conditions in China, oil-tea camellia trees have formed species with different growth habits, morphological characteristics, and degrees of oil quality [7]. *C. vietnamensis* is a species of oil-tea tree from Hainan Island, the southernmost city in China with a unique geographical location and superior climate [8], and some other tropical countries, such as Thailand and Vietnam [9]. *C. vietnamensis* from Hainan Island, which is considered an independent and traditional plant resource according to the long-term isolation from the mainland [7,10], is somewhat different from *C. oleifera*, which is widely grown in mainland China. It is more suitable for a tropical climate, has a large amount of genetic variation, and has higher contents of active ingredients in the oil [1,8].

Simple sequence repeats (SSRs), also called short tandem repeats (STRs) or microsatellites, are widely distributed in the genomes of animals and plants [11]. The random distribution of SSRs in the genome, together with the high level of allelic variation in microsatellite loci, makes them an ideal marker for studying population structure and genetic relationships [12,13]. Designing suitable genetic markers using SSRs allows detailed understanding of the composition and regulatory mechanisms of loci controlling quantitative or disease-resistance traits, allowing one to construct genetic linkage groups with genetic markers [13]. Further manipulation of these genes, the identification and cloning of QTLs affecting target traits, and studying the diversity of population genetics will assist with reaching the goal of marker-assisted selection of an improved population or genotypic selection of an improved population [14]. Several polymorphic SSR markers have been built and used to analyze population structure and genetic relationship in *Camellia* species [15], such as *Camellia sinensis* [11], *C. chekiangoleosa* [16], *C. oleifera* [17], *Camellia japonica* [15], and *Camellia fascicularis* [18]. Huang for the first time analyzed the inter-species hybrid introgression and genetic structure between *Camellia meiocarpa* and *C. oleifera* by SSR markers [19]. Combining morphological traits and SSR markers analysis, He et al. found that *C. oleifera* had abundant genetic variation [17]. An unidentified oil-tea *Camellia* species from Hainan was identified by the chloroplast genome sequences and SSR analysis [1]. As a consequence, it was feasible to study the population structure and genetic relationships of oil-tea camellia species using SSRs.

To date, molecular marker studies in *Camellia* species have mainly involved SSRs [11,17], RAPD [20,21]), ISSR [22,23], and so on. However, few SSRs studies have compared *C. vietnamensis* and other *Camellia* species. Therefore, in this study, we collected SSR molecular markers from 140 oil-tea camellia samples, followed by the non-hierarchical analysis of molecular variance (AMOVA), the unweighted pair group method with arithmetic (UPGMA), principal coordinates analysis (PCoA), and population structure analysis, in the hope of providing some data basis and theoretical basis for the delineation of the relatives, resource system, and population structure of oil-tea camellia species.

## 2. Materials and Methods

### 2.1. Materials

A collection of 140 oil-tea camellia accessions was used in this study, which were divided into 5 groups (Table 1), including 114 oil-tea camellia leaves and 26 oil-tea camellia seeds. The samples in this study were identified by Prof. Kaibing Zhou in 2018. Among them, the leaves included 95 *C. oleifera*, 17 *C. vietnamensis* species, and 2 *C. chekiangoleosa*. The seeds included 13 *C. oleifera*, 3 *C. chekiangoleosa*, 5 *C. crapnelliana*, and 5 *Camellia gauchowensis* specimens. Details of the samples are shown in Table 1 and Figure S1.

### 2.2. DNA Extraction

Sample DNA was extracted by the TIANGEN genomic DNA extraction kit (Beijing, China). DNA quality and concentration were then checked by 1% (*w/v*) agarose gel electrophoresis and the Agilent 2100 Bioanalyzer (USA). Good quality DNA was used directly for SSR analysis or stored at –20 °C for further use.

### 2.3. SSR Analysis

Ninety-six pairs of SSR primers were selected for pre-screening based on the transcriptome data of *C. vietnamensis* (NCBI accession number: PRJNA825399) [24], in which 15 fluorescently labeled SSR primers were selected for further research (Table 2). In addition, nine pairs of primers with good polymorphism were screened, referring to Song’s study (Table 2) [25]. The 5’ end of each forward primer for this analysis was labelled with FAM fluorescent dye (Applied Biosystems, USA). The M13 universal linker sequence (TGTAAAACGACGGCCAGT) was used to add to the 5’ direction of the forward primer of each pair of primers, and M13 linker sequences with different fluorescent groups were synthesized. Following the method of Gu [26] with minor modification, the SSR-PCR amplification was performed in a 15 µL total reaction volume, including 1.0 µL (5 pmol·µL^−1^) of forward and reverse primers, 7.5 µL of 2 × Taq PCR master mix (Gene tech, Shanghai, China), 1 µL (50 ng·µL^−1^) of template DNA, and 4.5 µL of ddH_2_O. The PCR program was as follows: 96 °C, 3 min; 96 °C for 30 s, 50–60 °C for 30 s, and 72 °C for 1 min, and these three procedures were cycled 30 times; 72 °C, 10 min. Two microliters of amplified PCR products were used in 2% (*w/v*) agarose gel electrophoresis to check whether the amplified fragment size and concentration were in the normal ranges at each locus with reference to the DNA marker alignment. Then, 1.0 µL of the fluorescent PCR product was diluted 30 fold with ultrapure water and prepared for machine detection. The diluted PCR products were separated by capillary electrophoresis by the ABI 3730XL DNA Analyzer (Applied Biosystems, Foster City, CA, USA), and data were handled by Gene Marker v.2.2.0 software (Soft Genetics, State College, PA, USA).

### 2.4. Data Acquisition and Analysis

According to the PCR results, a binary matrix was formed in which the presence of the product was marked as 1 and the absence of the product as 0. The results of the 1/0 data matrix were utilized to analyze the genetic diversity of oil-tea camellia trees. Based on the number of alleles, the level of discrimination of each SSR marker was assessed by calculating the percentage of polymorphic loci (P), Nei’s genetic diversity (h), Shannon diversity index (I) [27], gene differentiation coefficient (Gst) [26], and gene flow from Gst (Nm). Nei’s genetic diversity (h) and Shannon diversity index (I) were calculated using the POPGENE software [28].

According to the DICE coefficient [29], Nei’s genetic distance (D) and genetic identity between different groups were further calculated using GenAlex software [30]. The degrees of genetic variation among and within groups were analyzed by the non-hierarchical analysis of molecular variance (AMOVA) method, with 9999 random permutations [28]. Then, the unweighted pair group method with arithmetic (UPGMA) and the principal coordinates analysis (PCoA) were performed [31]. PCoA analysis was performed with GenAlex software. Linkage disequilibrium was analyzed using the pair.ia method of the R package poppr, and plots were drawn in R. In addition, the genetic structure of oil-tea camellia samples was analyzed by STRUCTURE [32], which is a model-based Bayesian clustering program with a range of genetic clusters from K = 3 to 10. Twenty independent runs were evaluated for each fixed K, and the best potential clusters (K value) were checked by the ∆K method on the STRUCTURE Harvester program [32]. The running results were integrated by CLUMPP software [33].

## 3. Results

### 3.1. Assessment of SSR Marker Diversity Levels

The 140 accessions from five oil-tea camellia species were analyzed by SSR markers. The alleles detected by 24 pairs of primers at the polymorphic sites ranged from 6 to 31. A total of 385 alleles were generated by amplification, resulting in an average of 16.0417 alleles per locus (Table 3). The mean of Nei’s gene diversity (h) and Shannon’s information index (I) were 0.1104 and 0.1890, which indicate that the genetic diversity was not very rich. It can be seen in Table 3 and Table S1 that the range of total genetic variation Ht was 0.0019–0.5000; the average value was 0.1153. The range of genetic variation within population Hs was 0–0.4601; the average value was 0.0698. The gene differentiation coefficient Gst value ranged from 0.0037 to 1.0000, and the average was 0.3948, indicating 39.48% genetic variation among individuals and a high degree of genetic differentiation. The range of gene flow (Nm) values of the whole population was 0–134.0618, and the average value was 0.7666, indicating that there was little gene exchange among the oil-tea camellia group. 

**Figure 1:**
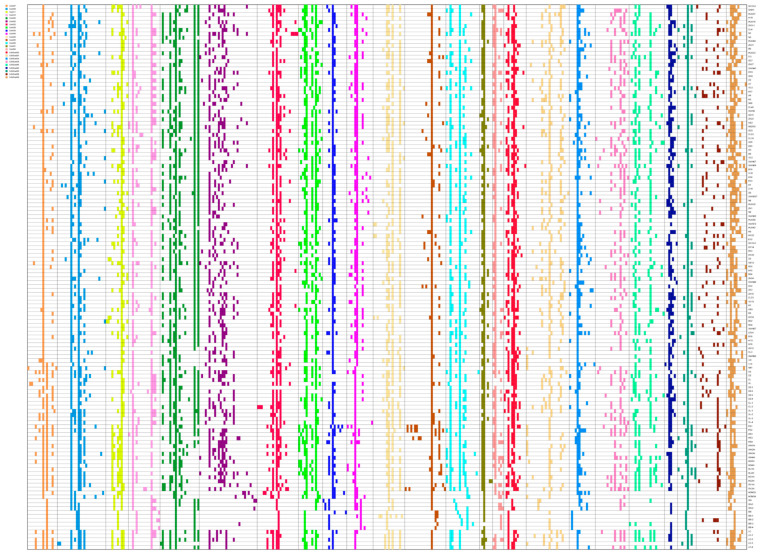
The fingerprinting of each allele in 140 samples.

The alleles at each locus in each sample were coded into a fingerprint in the form of a 0/1 matrix based on bands amplified using 24 pairs of primers. Fingerprinting gives a visual representation of the differences for each sample (Figure 1). As could be found from the fingerprinting of 140 oil-tea camellia accessions, these 24 pairs of primers could discriminate some of the 140 accessions.

### 3.2. Genetic Diversity of Oil-Tea Camellia Species Based on SSR Analysis

The detailed information of each genetic locus of each species is shown in Table S2. The average sample size was 28 for each species (Table 4). The mean Na was 0.735 (range: 0.200–1.605). The average Ne was 1.138 (range: 1.041–1.197). The mean h was 0.086 (range: 0.027–0.128), and the mean uh was 0.096. The average *I_s_* within species reached 0.134. S1 had the highest genetic variability (0.214), and S4 had the lowest value (0.041). When computed at the individual level, the mean *I* was 0.1890. The results indicate that the genetic differences among different groups were small and the genetic diversity was not very rich.

### 3.3. Analysis of Nei’s Genetic Distance between Species

**Figure 2:**
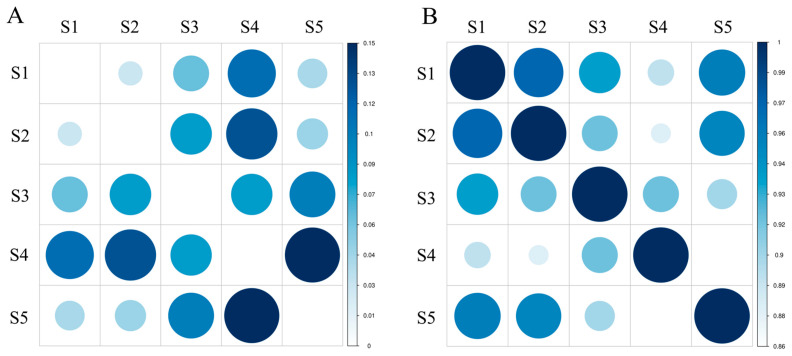
Genetic distance and genetic identity among groups. (**A**): Nei’s genetic distance; (**B**): Nei’s genetic identity.

Nei’s genetic distance (D) is a measure of genetic difference among biological populations and can be measured in terms of quality traits and also with quantitative traits. The estimation of genetic distance is important for exploring the origins of cultivars, analyzing the relationships among populations, mapping phylogenetic trees and predicting heterosis, and guiding parental selection. The range of genetic identity among species was 0.8616–0.9719, calculated from 285 amplified fragments. As shown in Figure 2, S1 and S2 had the smallest genetic distance (0.0285) and the largest genetic identity (0.9719) with the closest relatives, followed by S1 and S5. S5 and S4 had the largest genetic distance (0.1490), shared the least genetic identity (0.8616), and were the most distantly related, followed by S4 and S2. The results of AMOVA indicated that most of the genetic variation (77%) occurred within species and only a small fraction (23%) occurred among species (Table 5). In addition, there were significant differences within and among groups. The mean fixation index (*F*_st_) among five groups showed moderate genetic differentiation (*F*_st_ = 0.231).

### 3.4. UPGMA and PCoA Analysis

**Figure 3:**
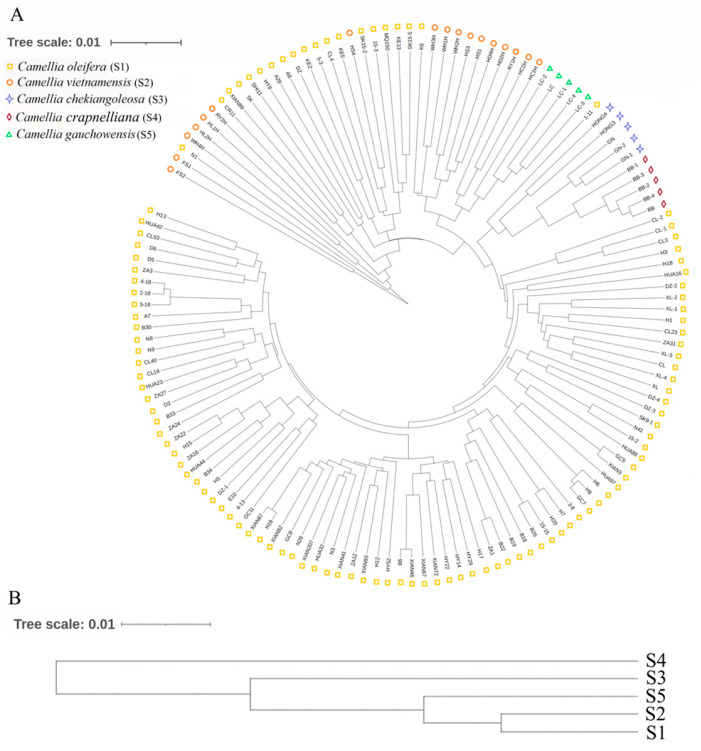
The UPGMA phylogenetic tree of oil-tea camellia samples based on SSR data. (**A**): The phylogenetic tree of 140 samples; (**B**): The phylogenetic tree of five *Camellia* species.

**Figure 4:**
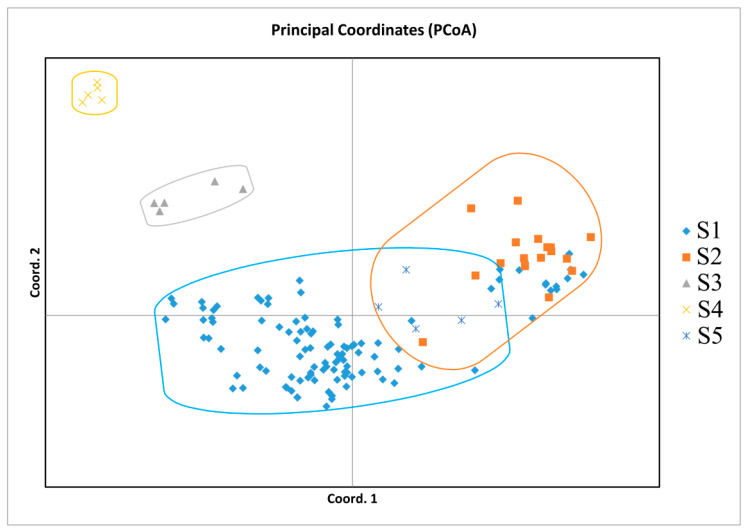
PCoA of oil-tea camellia samples based on SSR data.

Based on Nei’s genetic distances among individuals and groups, the clustering analysis among individuals was accomplished using the aboot method of the R package poppr, by selecting Nei’s distance and bootstrapping 1000 times. Cluster analysis among populations was subjected to UPGMA trees drawn using the phylip software. According to the genetic distances, a phylogenetic tree was built (Figure 3). As can be seen in Figure 3A, most individuals from S1 grouped together; S2, S5, and a small part of S1 were clustered together; individuals of S3 and S4 grouped together. The phylogenetic tree obtained with Nei’s genetic distance classified the species into three main clades (Figure 3B). The first clades included S1, S2, and S5; the other two were S3 and S4. Among them, *C. oleifera* was clustered into one subclade, *C. vietnamensis* and *C. crapnelliana* were clustered into one subclade, and *C. chekiangoleosa* and *C. gauchowensis* were clustered into one subclade. In addition, some *C. oleifera* and *C. vietnamensis* were clustered into one subclade. Furthermore, two-dimensional PCoA revealed four distinct clusters on the basis of Nei’s genetic distance among individuals (Figure 4). PCoA analysis reflects the variability between two samples or two groups by an intuitive comparison of the straight-line distances between samples in the coordinate axis, which indicates whether the two samples or two groups of samples are notably divergent. PCoA of the first three axes explained 17.61% of the total variation (7.30%, 6.68%, and 3.63%, respectively). The results of PCoA were relatively similar to the individual-based phylogenetic tree. S1 (*C. oleifera*) samples were clustered together, S2 (*C. vietnamensis*) and S5 (*C. gauchowensis*) samples were clustered together, S3 (*C. chekiangoleosa*) samples were clustered together, and S4 (*C. crapnelliana*) samples were clustered together.

### 3.5. Linkage Disequilibrium Analysis and Population Structure

**Figure 5:**
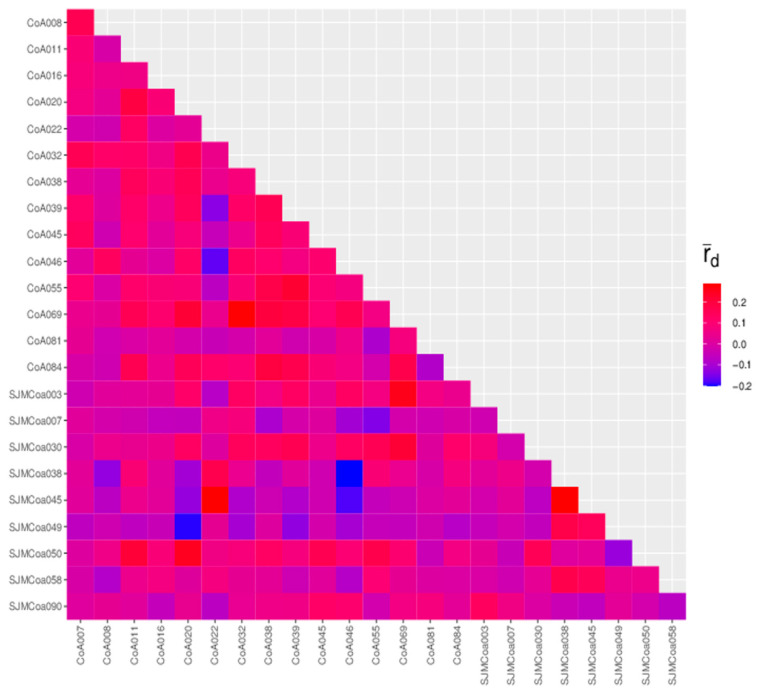
The linkage disequilibrium of 24 SSR loci.

In linkage disequilibrium, there is a shift between the probability that a haplotype will appear and the probability that it will be randomly combined. The extent of this offset determines the extent of linkage disequilibrium. The degree of linkage disequilibrium was characterized by the square of the R value, which, when equal to 0, indicates complete linkage equilibrium—independent inheritance. When the R-squared equals 1, it indicates complete linkage disequilibrium. All 24 SSR loci were in linkage disequilibrium with each other; the a maximum R-squared was 1, and a minimum R-squared was 0.0099 (Figure 5 and Table S3).

**Figure 6:**
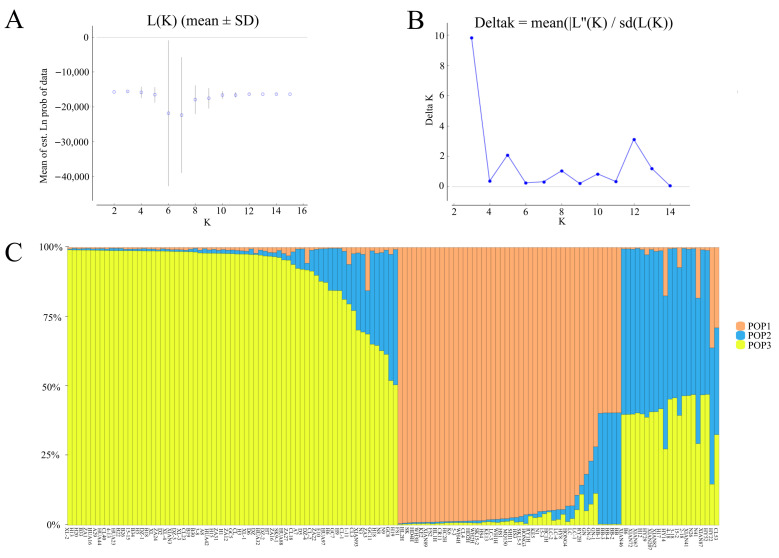
A structure analysis of 140 oil-tea camellia samples. (**A**): Estimated LnP(D) of K from 2 to 16. (**B**): ΔK according to the rate of change of LnP(D) between successive K. (**C**): Genetic structure of oil-tea camellia population.

The results of STRUCTURE showed a clear maximum for Ln(PD)-derived delta K (∆K) at K = 3 (Figure 6A,B), and this was considered as a possible number for the population of oil-tea camellia. Therefore, it indicated that the studied accessions belonged to three different clusters (Figure 6C). Among them, most of *C. oleifera* samples were clustered in one population; a small proportion of *C. oleifera* samples were clustered separately; *C. vietnamensis*, *C. gauchowensis*, *C. crapnelliana*, and *C. chekiangoleosa* were another cluster. The results of population structure analysis were similar to those of UPGMA analysis (Figure 3A).

## 4. Discussion

In this research, the genetic diversity of five oil-tea camellia species was analyzed by using SSR markers. The range of alleles in SSR was 6–31. A total of 385 alleles were found. An average of 16.0417 alleles were found for each SSR-primer pair. At the species level, the range of *Ip* of oil-tea camellia was 0.041–0.214, and the range of *p* was 7.79–79.48% (the mean value was 34.49%), showing moderate genetic diversity. When the polymorphism information content (PIC) was less than 0.25, SSR primers showed little polymorphism; when 0.25 < PIC < 0.5, moderate polymorphism; when PIC > 0.5, high polymorphism [34]. The results from the amplification of 345 pairs of SSR primers by Shi et al. [16] indicated that the proportion of polymorphic sites (31.9%) was relatively high. Chai et al. analyzed six natural populations of *C. pubipetala*, and the results showed that the I value was 0.4100; the PPB (percentage of polymorphic bands) was 80.43% [35]. Although the six populations’ distribution was narrow, the genetic diversity was high. A total of 495 alleles were identified by 111 SSR loci in *C. japonica*, and the range of alleles was 1–12. The mean was 4.46 alleles per locus. The range of PIC was 0.15–0.86, and the average was 0.59 [15]. The mean of *p* in this study was 34.49%, which is similar to the above results. The ranges of Ne, h, and *Ip* of 24 markers in this study were 1.041–1.197, 0.027–0.128, and 0.041–0.214, respectively. The differences are larger when compared with the results of Dong et al., who used 16 SSR marker pairs for 54 oil-tea trees (including *C. polyodonta*, *C. oleifera*, *C. gauchowensis*, and *C. semiserrata*) for genetic diversity analysis. The ranges of Ne, h, and I were 1.17–1.70, 0.14–0.40, and 0.26–0.59, respectively [3]. In conclusion, the SSR primers in this study showed moderate to high levels of polymorphism, which indicates that they were suitable for genetic diversity analysis of oil-tea camellia trees.

Accurate genetic relationships among germplasm accessions are important for variety development, evolutionary studies, and resource conservation [31,36]. Three main clusters were determined by the UPGMA method on 140 samples. *C. vietnamensis* was clustered with *C. gauchowensis*, which is similar to the findings of Qi et al. [37] and Chen et al. [1]. They found that various indexes of leaf, flower, fruit, and seed morphologies of *C. vietnamensis* collected from Hainan Province showed high similarity to those of *C. gauchowensis*, whose provenance was Gaozhou in Guangdong Province [37], and *C. vietnamensis* and *C. gauchowensis* were found to be clustered together by cpDNA sequences and SSR marker analysis [1], so it could be speculated that the relative proximity of *C. vietnamensis* and *C. gauchowensis* to each other was quite near. Dai et al. analyzed the chloroplast genome trnH-psbA and matK sequences of 101 different kinds of oil-tea camellia seedlings by DNA barcoding technology [38]. They found that *C. vietnamensis* was clustered into one branch and *C. chekiangoleosa* was clustered into another, and the clustering results in this study agree strongly with these results. The findings suggest that *C. vietnamensis* in Hainan has a relatively close relative. Additionally, the phenomenon of self-incompatibility might occur in close relatives, which might be one of the reasons for the low seed-setting rate of *C. vietnamensis* in Hainan. 

For a more accurate analysis of the genetic structure of oil-tea camellia, STRUCTURE was used for further analysis, and the results indicated that the 140 accessions were classified into three clusters. Among them, most of *C. oleifera* samples were clustered one population; a small proportion of *C. oleifera* were in another cluster; and *C. vietnamensis*, *C. gauchowensis*, *C. crapnelliana*, and *C. chekiangoleosa* were one cluster. There were small fractions of *C. oleifera* samples that clustered with other *Camellia* species. It was indicated that plants of the same group came not only from the same region but also from different regions. Possibly, species with different genetic backgrounds may cluster together. This indicates that the kinship of germplasm is extremely complex. The reasons for this phenomenon might be as follows. First, occasional genetic mutations and long-term natural selection have made finding the relatives of oil-tea camellia more complicated. Second, the effect of genetic drift was greater during the natural differentiation of oil-tea camellia than those of natural environmental factors, leading to the failure to divide by geographic region when clustering. Using indirect measures, the gene flow among populations was estimated by the value of Nm [28]. The Nm value (0.7666) indicated low gene flow among species and might promote population differentiation. When Nm < 1, genetic drift is thought to be a major contributor to population differentiation [26,28]. Third, the uneven number of selected samples makes the clustering result not accurate enough, and so on.

The results of genetic structure analysis indicate that the genetic variation of oil-tea camellia samples mainly appeared within species, accounting for 77% of the total variation, leaving only a small portion (23%) occurring among species. That might result from habitat fragmentation and geographical barriers. Some experts have also obtained similar results with other *Camellia* plants. He et al. used nine pairs of SSR primers to analyze 150 accessions of *C. oleifera*, and the results indicated that the genetic diversity level of *C. oleifera* is high [17]. In addition, Li et al. also analyzed 84 accessions of eight natural populations of *C. fascicularis* with fourteen pairs of primers for SSR markers [18]. The results indicated that the eight populations of *C. fascicularis* were roughly divided into three clusters, and the genetic variation within populations accounted for 49.95% of variation. To sum up, the results of this study indicate that the genetic variation of oil-tea camellia samples was mainly found within populations, and inbreeding occurred within the population, such as with *C. vietnamensis*. The degree of gene exchange among species was low.

## 5. Conclusions

In this research, 24 pairs of SSR primers were selected to analyze the genetic relationship and population structure of 140 oil-tea camellia accessions using fluorescence detection by capillary electrophoresis. The results indicate that genetic diversity was abundant among the 140 *Camellia* accessions. Based on genetic distances and clustering by UPGMA, the 140 accessions could be classified into three clusters. Most individuals from S1 grouped together, samples from S2 and S5 grouped together, and samples from S3 and S4 formed the same branch. In addition, some individuals from S1 and S2 were clustered together, which relates to the results of the PCoA. The Bayesian-model-based genetic structure analysis indicated that the studied accessions belonged to three populations. Among them, most of the *C. oleifera* samples were clustered into one population; a small proportion of *C. oleifera* were in another cluster; *C. vietnamensis*, *C. gauchowensis*, *C. crapnelliana*, and *C. chekiangoleosa* were one cluster. Taken together, the findings should be instructive for oil-tea camellia species’ introduction, breeding, germplasm preservation, and new-variety development, and provide a theoretical foundation for the classification and identification of oil-tea camellia species in southern China and the research on relatives.

## Figures and Tables

**Table 1:** Detailed information for the 140 oil-tea camellia accessions used in this study.

No.	Name	Tissue	species	Group	Origin	Location
1	1_11	Leaves	*C. oleifera*	S1	Nursery of oil-tea camellia germplasm resources, Danzhou campus, Hainan University, China	109°29′45″ E, 19°30′28″ N
2	15_15		*C. oleifera*	S1		
3	15_2		*C. oleifera*	S1		
4	15_3		*C. oleifera*	S1		
5	2_18		*C. oleifera*	S1		
6	3_18		*C. oleifera*	S1		
7	3_8		*C. oleifera*	S1		
8	4_13		*C. oleifera*	S1		
9	4_18		*C. oleifera*	S1		
10	5_3		*C. oleifera*	S1		
11	A29		*C. oleifera*	S1		
12	A7		*C. oleifera*	S1		
13	A8		*C. oleifera*	S1		
14	B18		*C. oleifera*	S1		
15	B19		*C. oleifera*	S1		
16	B22		*C. oleifera*	S1		
17	B26		*C. oleifera*	S1		
18	B30		*C. oleifera*	S1		
19	B33		*C. oleifera*	S1		
20	B34		*C. oleifera*	S1		
21	B6		*C. oleifera*	S1		
22	CL18		*C. oleifera*	S1		
23	CL23		*C. oleifera*	S1		
24	CL3		*C. oleifera*	S1		
25	CL40		*C. oleifera*	S1		
26	CL4		*C. oleifera*	S1		
27	CL53		*C. oleifera*	S1		
28	CR11		*C. oleifera*	S1		
29	D2		*C. oleifera*	S1		
30	D5		*C. oleifera*	S1		
31	D6		*C. oleifera*	S1		
32	E10		*C. oleifera*	S1		
33	GC11		*C. oleifera*	S1		
34	GC5		*C. oleifera*	S1		
35	GC7		*C. oleifera*	S1		
36	GC8		*C. oleifera*	S1		
37	H12		*C. oleifera*	S1		
38	H13		*C. oleifera*	S1		
39	H15		*C. oleifera*	S1		
40	H17		*C. oleifera*	S1		
41	H18		*C. oleifera*	S1		
42	H19		*C. oleifera*	S1		
43	H1		*C. oleifera*	S1		
44	H20		*C. oleifera*	S1		
45	H3		*C. oleifera*	S1		
46	H5		*C. oleifera*	S1		
47	H6		*C. oleifera*	S1		
48	H7		*C. oleifera*	S1		
49	H9		*C. oleifera*	S1		
50	HUA16		*C. oleifera*	S1		
51	HUA23		*C. oleifera*	S1		
52	HUA32		*C. oleifera*	S1		
53	HUA42		*C. oleifera*	S1		
54	HUA44		*C. oleifera*	S1		
55	HUA88		*C. oleifera*	S1		
56	HUA97		*C. oleifera*	S1		
57	HY14		*C. oleifera*	S1		
58	HY22		*C. oleifera*	S1		
59	HY29		*C. oleifera*	S1		
60	HY52		*C. oleifera*	S1		
61	HY8		*C. oleifera*	S1		
62	K6		*C. oleifera*	S1		
63	KE13		*C. oleifera*	S1		
64	KE2		*C. oleifera*	S1		
65	KE5		*C. oleifera*	S1		
66	MQ150		*C. oleifera*	S1		
67	N1		*C. oleifera*	S1		
68	N26		*C. oleifera*	S1		
69	N3		*C. oleifera*	S1		
70	N41		*C. oleifera*	S1		
71	N8		*C. oleifera*	S1		
72	N9		*C. oleifera*	S1		
73	SHI11		*C. oleifera*	S1		
74	SK15-2		*C. oleifera*	S1		
75	SK15-5		*C. oleifera*	S1		
76	SK9-1		*C. oleifera*	S1		
77	SK		*C. oleifera*	S1		
78	XIAN207		*C. oleifera*	S1		
79	XIAN3		*C. oleifera*	S1		
80	XIAN41		*C. oleifera*	S1		
81	XIAN46		*C. oleifera*	S1		
82	XIAN67		*C. oleifera*	S1		
83	XIAN72		*C. oleifera*	S1		
84	XIAN82		*C. oleifera*	S1		
85	XIAN87		*C. oleifera*	S1		
86	XIAN89		*C. oleifera*	S1		
87	XIAN93		*C. oleifera*	S1		
88	ZA12		*C. oleifera*	S1		
89	ZA16		*C. oleifera*	S1		
90	ZA1		*C. oleifera*	S1		
91	ZA22		*C. oleifera*	S1		
92	ZA24		*C. oleifera*	S1		
93	ZA27		*C. oleifera*	S1		
94	ZA31		*C. oleifera*	S1		
95	ZA3		*C. oleifera*	S1		
96	FS1		*C. vietnamensis*	S2	Fansai Village, Wuzhishan City, Hainan Province	109°32′24″ E, 18°50′37″ N
97	FS2		*C. vietnamensis*	S2		
98	HC1H		*C. vietnamensis*	S2	Fushan Town, Chengmai County, Hainan Province	109°54′55″ E, 19°52′20″ N
99	HC2H		*C. vietnamensis*	S2		
100	HD2H		*C. vietnamensis*	S2	Shangke Town, Qionghai City, Hainan Province	110°20′39″ E, 19°04′20″ N
101	HD4H		*C. vietnamensis*	S2		
102	HL1H		*C. vietnamensis*	S2	Qiongshan Area, Haikou City, Hainan Province	110°21′54″ E, 19°59′25″ N
103	HL2H		*C. vietnamensis*	S2		
104	HS1		*C. vietnamensis*	S2	Hongshan Village, Wuzhishan City, Hainan Province	109°30′56″ E, 18°51′35″ N
105	HS3		*C. vietnamensis*	S2		
106	HS4		*C. vietnamensis*	S2		
107	RY1H		*C. vietnamensis*	S2	Wencheng Town, Wenchang City, Hainan Province	110°47′38″ E, 19°33′13″ N
108	RY2H		*C. vietnamensis*	S2		
109	WH1H		*C. vietnamensis*	S2	Wanling Town, Qiongzhong County, Hainan Province	109°53′48″ E, 19°08′35″ N
110	WH2H		*C. vietnamensis*	S2		
111	WH3H		*C. vietnamensis*	S2		
112	WH4H		*C. vietnamensis*	S2		
113	HONG3		*C. chekiangoleosa*	S3	Wuzhishan City, Hainan Province	109°30′57″ E, 18°46′29″ N
114	HONG4		*C. chekiangoleosa*	S3		
115	CL	Seeds	*C. oleifera*	S1	Xixiangtang Area, Nanning City, Guangxi Zhuang Autonomous Region	108°21′7″ E, 22°55′6″ N
116	CL-1		*C. oleifera*	S1		
117	CL-2		*C. oleifera*	S1		
118	DZ		*C. oleifera*	S1		
119	DZ-1		*C. oleifera*	S1		
120	DZ-2		*C. oleifera*	S1		
121	DZ-3		*C. oleifera*	S1		
122	DZ-4		*C. oleifera*	S1		
123	XL		*C. oleifera*	S1		
124	XL-1		*C. oleifera*	S1		
125	XL-2		*C. oleifera*	S1		
126	XL-3		*C. oleifera*	S1		
127	XL-4		*C. oleifera*	S1		
128	GN		*C. chekiangoleosa*	S3		
129	GN-1		*C. chekiangoleosa*	S3		
130	GN-2		*C. chekiangoleosa*	S3		
131	BB		*C. crapnelliana*	S4		
132	BB-1		*C. crapnelliana*	S4		
133	BB-2		*C. crapnelliana*	S4		
134	BB-3		*C. crapnelliana*	S4		
135	BB-4		*C. crapnelliana*	S4		
136	LC		*C. gauchowensis*	S5		
137	LC-1		*C. gauchowensis*	S5		
138	LC-2		*C. gauchowensis*	S5		
139	LC-3		*C. gauchowensis*	S5		
140	LC-4		*C. gauchowensis*	S5		

**Table 2:** Detailed information for 24 pairs of primers in the study.

No.	Locus	Repeat Unit	Forward Sequence	Reverse Sequence	Pre Experiment Size (bp)	Fluorescent Dyes
1	CoA007	(TCT)6	CCAATCTCCAAACGCAACTT	CAGAGGAAATCGAGAGGCAG	245	FAM
2	CoA008	(ATAG)6	CCAGCCAGCTAAGAGGTTTG	CAGGTCATAGCTACCACGGA	188	FAM
3	CoA011	(CTT)5	TGGGTGGCTCAATATCATCA	ACCGGCCATTTATATGGGTT	200	FAM
4	CoA016	(ATC)6	GTAAGTCTCTGCACCGCCTC	TCGATTTCGTCCAATCCTTC	211	FAM
5	CoA020	(AGG)6	AGGGCATAAGAGGGAGTGGT	CGACCTCGACCTTCAAGAAC	207	FAM
6	CoA022	(GA)12	TAGCCAATAACATGCCCACA	AGTTGTCCAACCCTTCCTCA	147	FAM
7	CoA032	(GCG)5	TTATTCTTCGGGAACAACGG	ACACATGAAACAACGGCAAA	170	FAM
8	CoA038	(GTG)7	GAGATCGGCCAGAGTTTGAG	CATCAAAGCCACACTCGCTA	202	FAM
9	CoA039	(TTA)6	GCAAGAGGTCTCTTTGGGTG	AACCTCATGAGCTAAAGCCG	113	FAM
10	CoA045	(ACC)5	TCCAAACAGGCCAACTAAGC	GCTTGAGAAACCCAAAGCAG	244	FAM
11	CoA046	(TAAC)4	AACCAGAGGAACATCCAACG	TATCCTTGCCGCTTTGAATC	196	FAM
12	CoA055	(CAT)6	TCTGGTGTGCTTCAAACTGC	GCTCCAGCAAATATTCAGGC	265	FAM
13	CoA069	(TGC)6	CATGGCTTGGCTTCAATCTT	CAATGTTCCCAAGCGATTCT	224	FAM
14	CoA081	(CAA)5	ATATGAATCGGCCAATCGAC	AGATGACGCCTTTCGAAGAA	154	FAM
15	CoA084	(GTG)6	GACGGCTTAAACATGGAGGA	TTCATTTAATGGCAGGAGGC	110	FAM
16	SJMCoa003	(CAA)7	ACGAAACATGTCGGACGTGA	GGGAATGGACGAGACTTGGG	120	FAM
17	SJMCoa007	(TTC)6	GCAGCAGCGAGAGTAACAGT	GTGGGACGATTGAGCTTCCT	149	FAM
18	SJMCoa030	(CCT)10	GGTGTGGTGGTGAAGCAGTA	TTGTCTGGATCCATAGCCGC	248	FAM
19	SJMCoa038	(TTAT)5	TGCTTGGTCACTACCCAGTC	TGACACCTTGGTGCCAAAGA	266	FAM
20	SJMCoa045	(AAT)5	TTTGGGCGGGCAAAGATTTG	ACTCAAGCATGGACATCGGG	276	FAM
21	SJMCoa049	(AAT)5	AAGACCCAAACTGGACTGCA	ACCTTGCACCATAATGGGTT	254	FAM
22	SJMCoa050	(AAT)7	TGGAGCGTTAGTCTGGAGTC	GGCCTCTCATCCATGTCAGG	249	FAM
23	SJMCoa058	(CCA)9	GTGCCCTGTGACACCAAGTA	CGACGGTGGAGATTTGGTGA	245	FAM
24	SJMCoa090	(TCA)9	ACAGAAGGCGTTTGAGTCAA	GGCTTCTTCTTCGGAACCCA	165	FAM

**Table 3:** Genetic parameters of the SSR locus analysis.

Locus	Product Size (bp)	Number of Alleles	Ne	h	*I*	Gst	Nm
CoA007	176–257	16	1.0072–1.6880	0.0071–0.4076	0.0237–0.5976	0.0037–0.7133	0.2010–134.0618
CoA008	138–230	26	1.0072–1.7521	0.0071–0.4293	0.0237–0.6206	0.0037–0.7981	0.0029–134.0618
CoA011	167–206	12	1.0072–1.5542	0.0071–0.3566	0.0237–0.5393	0.0037–1.0000	0–134.0618
CoA016	208–367	17	1.0072–1.7183	0.0071–0.3833	0.0237–0.6088	0.0037–0.7853	0.1367–134.0618
CoA020	164–256	21	1.0072–2.0000	0.0071–0.5000	0.0237–0.6931	0.0037–0.6424	0.2783–134.0618
CoA022	127–184	31	1.0072–1.9468	0.0071–0.4863	0.0237–0.6794	0.0037–0.7032	0.2110–134.0618
CoA032	130–204	22	1.0072–1.9872	0.0071–0.4968	0.0237–0.6899	0.0037–0.6555	0.2628–134.0618
CoA038	192–221	13	1.0072–1.9619	0.0071–0.4903	0.0237–0.6834	0.0037–0.6220	0.3038–134.0618
CoA039	103–126	13	1.0073–1.5290	0.0072–0.3460	0.0240–0.4973	0.0162–0.6118	0.3172–30.4262
CoA045	237–288	14	1.0072–1.9993	0.0071–0.4998	0.0237–0.6930	0.0037–0.7650	0.1536–134.0618
CoA046	169–208	17	1.0072–1.9983	0.0071–0.4996	0.0237–0.6927	0.0037–0.5994	0.3126–134.0618
CoA055	151–313	22	1.0073–1.2904	0.0072–0.2250	0.0240–0.3849	0.0112–1.0000	0–44.0603
CoA069	211–266	18	1.0072–1.9155	0.0071–0.4779	0.0237–0.6709	0.0037–0.8486	0.1211–134.0618
CoA081	150–184	7	1.0073–1.9835	0.0072–0.4959	0.0240–0.6890	0.0112–0.5857	0.3537–44.0603
CoA084	106–119	6	1.0072–1.6058	0.0071–0.3773	0.0237–0.5648	0.0037–0.4816	0.5381–134.0618
SJMCoa003	126–167	12	1.0072–1.9971	0.0071–0.4993	0.0237–0.6924	0.0037–0.6596	0.2581–134.0618
SJMCoa007	224–311	23	1.0072–1.9989	0.0071–0.4997	0.0237–0.6929	0.0037–0.4972	1.0755–134.0618
SJMCoa030	238–277	14	1.0072–1.7639	0.0071–0.4331	0.0237–0.6246	0.0037–1	0–134.0618
SJMCoa038	273–304	18	1.0072–1.8695	0.0071–0.4651	0.0237–0.6578	0.0037–0.2993	1.1705–134.0618
SJMCoa045	291–317	19	1.0073–1.9215	0.0072–0.4796	0.0240–0.6726	0.0075–0.3244	1.0415–66.5610
SJMCoa049	270–286	7	1.0072–1.9989	0.0071–0.4997	0.0237–0.6929	0.0037–0.3559	0.9048–134.0618
SJMCoa050	253–273	10	1.0072–1.3412	0.0071–0.2544	0.0237–0.4219	0.0037–1.0000	0–134.0618
SJMCoa058	197–266	16	1.0072–1.5438	0.0071–0.3522	0.0237–0.5371	0.0037–0.2238	1.7344–134.0618
SJMCoa090	173–203	11	1.0218–1.9829	0.0213–0.4957	0.0596–0.6888	0.0112–0.5934	0.3426–44.0603
Mean		16.0417	1.1676	0.1104	0.1890	0.3948	0.7666

Note: Ne, Number of Effective Alleles; h, Nei’s gene diversity; I, Shannon’s Information Index; Gst, Gene differentiation coefficient; Nm, estimate of gene flow from Gst.

**Table 4:** The population average diversity index.

Group	N	Na	Ne	*I*_s_	h	uh	P (%)
S1	108	1.605	1.197	0.214	0.128	0.130	79.48
S2	17	0.969	1.195	0.193	0.121	0.122	45.97
S3	5	0.434	1.140	0.119	0.081	0.101	20.52
S4	5	0.200	1.041	0.041	0.027	0.033	7.79
S5	5	0.468	1.116	0.104	0.070	0.087	18.70
Mean	28	0.735	1.138	0.134	0.086	0.096	34.49

Note: N, Sample size; Na, Number of different alleles; Ne, Number of effective alleles; Ip, intra-specie diversity; h, Nei’s gene diversity; uh, Unbiased diversity; P, Percentage of Polymorphic Loci.

**Table 5:** An analysis of molecular variance among and within *Camellia* species.

Variation Source	df	SS	MS	Est. Var.	PMV (%)	*F*_st_	*p* value
Among Pops	4	485.260	121.315	7.197	23	0.231	0.001
Within Pops	135	3240.862	24.006	24.006	77		
Total	139	3726.121		31.203	100		

Note: df, degree of freedom; SS, Square deviation; MS, Mean square deviation; Est. Var., Exist variance; PMV, Percentages of molecular variance; *F*_st_, coefficient of genetic differentiation. *p* value indicated significant differences of *p* ≤ 0.001.

## Data Availability

Not applicable.

## Supplementary Materials

The following supporting information can be downloaded at: https://www.mdpi.com/article/10.3390/genes13112162/s1, Table S1: Genetic diversity parameters of each SSR locus; Table S2: Genetic diversity parameters of each SSR locus of each specie; Table S3: Linkage disequilibrium of 24 SSR loci.
