# Supplementary material for: Assessment of the Genetic Relationship and Population Structure in Oil-Tea Camellia Species Using Simple Sequence Repeat (SSR) Markers
Source: Genes (Basel). 2022 Nov 19;13(11):2162. doi: 10.3390/genes13112162 (PMC9691144; doi:10.3390/genes13112162)
Supplement: Supplementary file 1 [file genes-13-02162-s001.zip › Supplementary figures.pdf]

Attached Figures

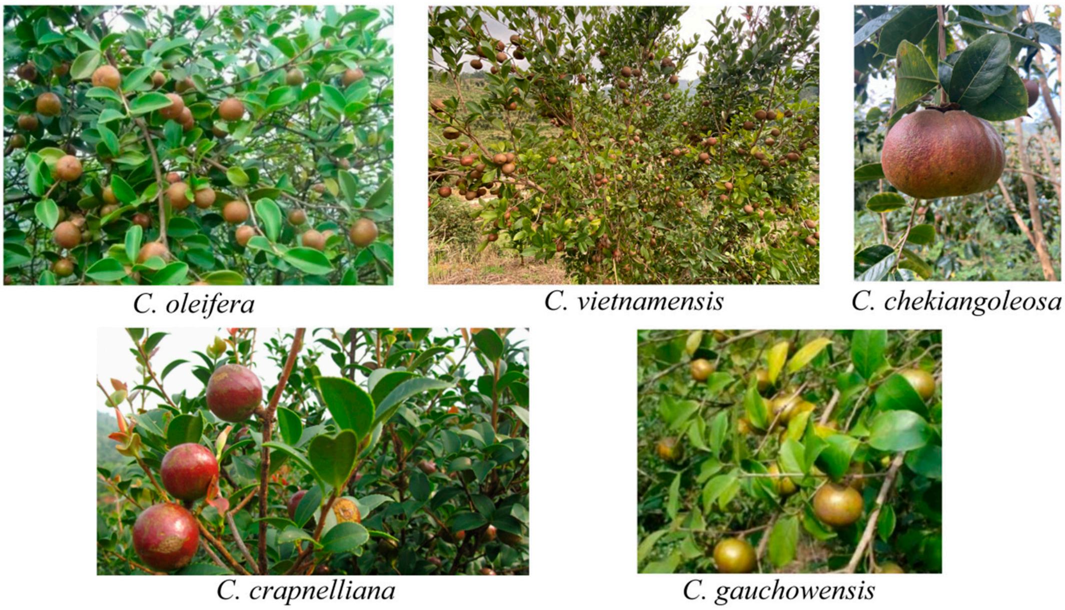

Figure S1. The general morphology view of five studied species.
